# Mapping trends and hotspots of mitochondrial dysfunction in Alzheimer’s disease from 2013 to 2022: a bibliometric analysis of global research

**DOI:** 10.3389/fnins.2023.1199625

**Published:** 2023-06-26

**Authors:** Wang Guo, Liban Abdulle Hassan, Yu-hao Chu, Xue-ping Yang, Sheng-xue Wang, Han-xiao Zhu, Yun Li

**Affiliations:** ^1^Clinical Medical School, Dali University, Dali, China; ^2^Department of Neurology, The First Affiliated Hospital of Dali University, Dali, China

**Keywords:** Alzheimer’s disease, mitochondrial dysfunction, bibliometric, VOSview, CiteSpace, Web of Science

## Abstract

**Objective:**

Alzheimer’s disease (AD), a prevalent neurodegenerative affliction that predominantly affects the elderly population, imposes a substantial burden on not only patients but also their families and society at large. Mitochondrial dysfunction plays an important role in its pathogenesis. In this study, we conducted a bibliometric analysis of research on mitochondrial dysfunction and AD over the past 10 years, with the aim of summarizing current research hotspots and trends in this field.

**Methods:**

On February 12, 2023, we searched for publications about mitochondrial dysfunction and AD in the Web of Science Core Collection database from 2013 to 2022. VOSview software, CiteSpace, SCImago, and RStudio were used to analyze and visualize countries, institutions, journals, keywords, and references.

**Results:**

The number of publications on mitochondrial dysfunction and AD were on the rise until 2021 and decreased slightly in 2022. The United States ranks first in the number of publications, H-index, and intensity of international cooperation in this research. In terms of institutions, Texas Tech University in the United States has the most publications. The *Journal of Alzheimer’s Disease* has the most publications in this field of research, while *Oxidative Medicine and Cellular Longevity* have the highest number of citations. Mitochondrial dysfunction is still an important direction of current research. Autophagy, mitochondrial autophagy, and neuroinflammation are new hotspots. The article from Lin MT is the most cited by analyzing references.

**Conclusion:**

Research on mitochondrial dysfunction in AD is gaining significant momentum as it provides a crucial research avenue for the treatment of this debilitating condition. This study sheds light on the present research trajectory concerning the molecular mechanisms underlying mitochondrial dysfunction in AD.

## Introduction

With the improvement of medical technology and the optimization of lifestyle, the average life span of humans is gradually increasing, but it also brings the problem of population aging. AD is a common disease among the elderly. It is a class of irreversible degenerative diseases with progressive cognitive dysfunction, memory loss, and behavioral changes (Maccioni et al., 2018). Eventually, the patient will lose basic living capacity and die (Alzheimer’s Association, 2020). There are approximately 50 million Alzheimer’s patients worldwide (Zhang et al., 2021), with a gradual increase of 5 million new cases per year (Schwab et al., 2022), and it will exceed 80 million by 2050 (Du et al., 2021). The number of patients with AD is increasing markedly worldwide. Today, the United States has approximately 6.5 million Alzheimer’s patients, which could reach 13.8 million by 2060 (Alzheimer’s Association, 2022). As a severely aging country, the number of patients with AD in Japan will reach 6.5–7 million by 2025 (Lv et al., 2023). A cross-sectional study from China showed that Alzheimer’s patients are 9.39–10.29 million people over 65 years of age, with an incidence of approximately 3.9% (Jia et al., 2020). Studies have shown that among Alzheimer’s patients over the age of 65, women were twice as likely as men (Alzheimer’s Association, 2014). Interestingly, data from Europe showed that men with AD had a higher mortality rate than women (Niu et al., 2017). A previous estimate suggested it could be the seventh leading cause of death in developed countries by 2030 (Mathers and Loncar, 2006). All these indicate that AD has become a serious burden to patients, families, and society. The pathogenesis of this disease is still not fully understood. Currently, the main hypotheses included β-amyloid protein (Aβ) deposition, the formation of neurofibrillary tangles caused by hyperphosphorylated tau protein, cerebrovascular dysfunction, and mitochondrial dysfunction (Yarns et al., 2022; Yu et al., 2022). Reports have shown that obvious energy metabolism disorders occurred in an AD brain, and mitochondrial dysfunction plays an important role in this situation, and this dysfunction often occurs before the onset of the disease (Yao et al., 2009; Mencer et al., 2021). Mitochondrial dysfunction leads to increased production of reactive oxygen species, apoptosis of vascular endothelial cells, and damage to the blood–brain barrier (Han et al., 2015). As the main place of energy production in cells, its metabolic dysfunction has been widely researched in various diseases of the nervous system, especially AD. At present, a large number of studies have shown that mitochondrial dysfunction promotes the pathological process of AD (Burte et al., 2015; Knuppertz et al., 2017). Currently, the research trends, details, and hot spots among AD and mitochondrial dysfunction are less reported.

Bibliometrics is a tool for scientific and technological evaluation; it can qualitatively and quantitatively evaluate scientific research results (Chen et al., 2014). It can select articles in a certain research field, conducting a comprehensive analysis of the country, author, keywords, publishing agency, journal, citation, and other topics. This method can scientifically evaluate the contributions of researchers, journals, and publishing institutions and also discover current research hotspots by analyzing keywords. Remarkably, it can visualize current research priorities and predict future research directions. This study used works of research in the Web of Science (WOS) as a data source to comprehensively analyze the research progress of mitochondrial dysfunction in AD through bibliometrics. Moreover, future research directions and hotspots of this field are presented by visualization.

## Materials and methods

### Data sources

**Figure 1:**
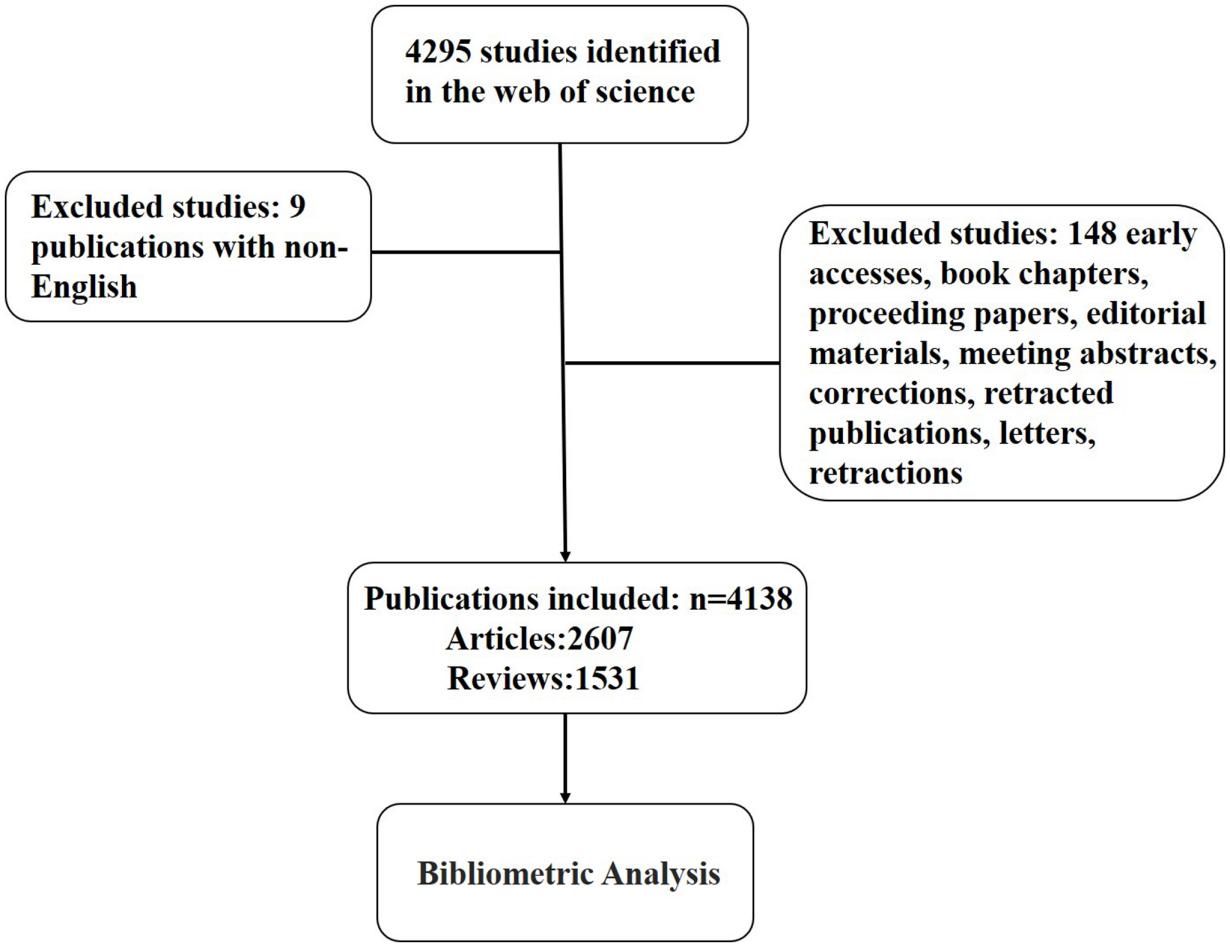
The flow chart of the research process.

The raw data for this study came from the Web of Science Core Collection database, where we selected all studies published between 01/01/2013 and 31/12/2022. Our studies included articles and reviews only, and the language was limited to English. To avoid changes in the included articles due to database updates, our search date is limited to 12/02/2023. The screening process was done independently by two researchers. Our solution formula: TS = [Alzheimer Disease OR Alzheimer Dementia OR Alzheimer Dementias OR Dementia, Alzheimer OR Alzheimer’s Disease OR Dementia, Senile OR Senile Dementia OR Dementia, Alzheimer Type OR Alzheimer Type Dementia OR Alzheimer-Type Dementia (ATD) OR Alzheimer Type Dementia (ATD) OR Dementia, Alzheimer-Type (ATD) OR Alzheimer Type Senile Dementia OR Primary Senile Degenerative Dementia OR Dementia, Primary Senile Degenerative OR Alzheimer Sclerosis OR Sclerosis, Alzheimer OR Alzheimer Syndrome OR Alzheimer’s Diseases OR Alzheimer Diseases OR Alzheimer’s Diseases OR Senile Dementia, Alzheimer Type OR Acute Confusional Senile Dementia OR Senile Dementia, Acute Confusional OR Dementia, Presenile OR Presenile Dementia OR Alzheimer Disease, Late Onset OR Late Onset Alzheimer Disease OR Alzheimer’s Disease, Focal Onset OR Focal Onset Alzheimer’s Disease OR Familial Alzheimer Disease (FAD) OR Alzheimer Disease, Familial (FAD) OR Familial Alzheimer Diseases (FAD) OR Alzheimer Disease, Early Onset OR Early Onset Alzheimer Disease OR Presenile Alzheimer Dementia] AND TS = (mitochondrial dysfunction). The specific flow chart of screening is shown in Figure 1.

### Data processing

The data from the Web of Science Core Collection database were selected and downloaded in txt. The downloaded data include information such as author, title, country, keywords, institution, journal, publication date, etc. We used Microsoft Excel 2019, GraphPad Prism 9.2.0, VOSviewer version 1.6.18, CiteSpace version 6.1. R6, SCImago Graphica Beta 1.0.27, and RStudio4.2.2 to present, analyze, and describe data.

### Bibliometric analysis

This paper has comprehensively described and analyzed the research topics, using countries, institutions, journals, authors, impact factors (IF), keywords, and H-index.

**Figure 2:**
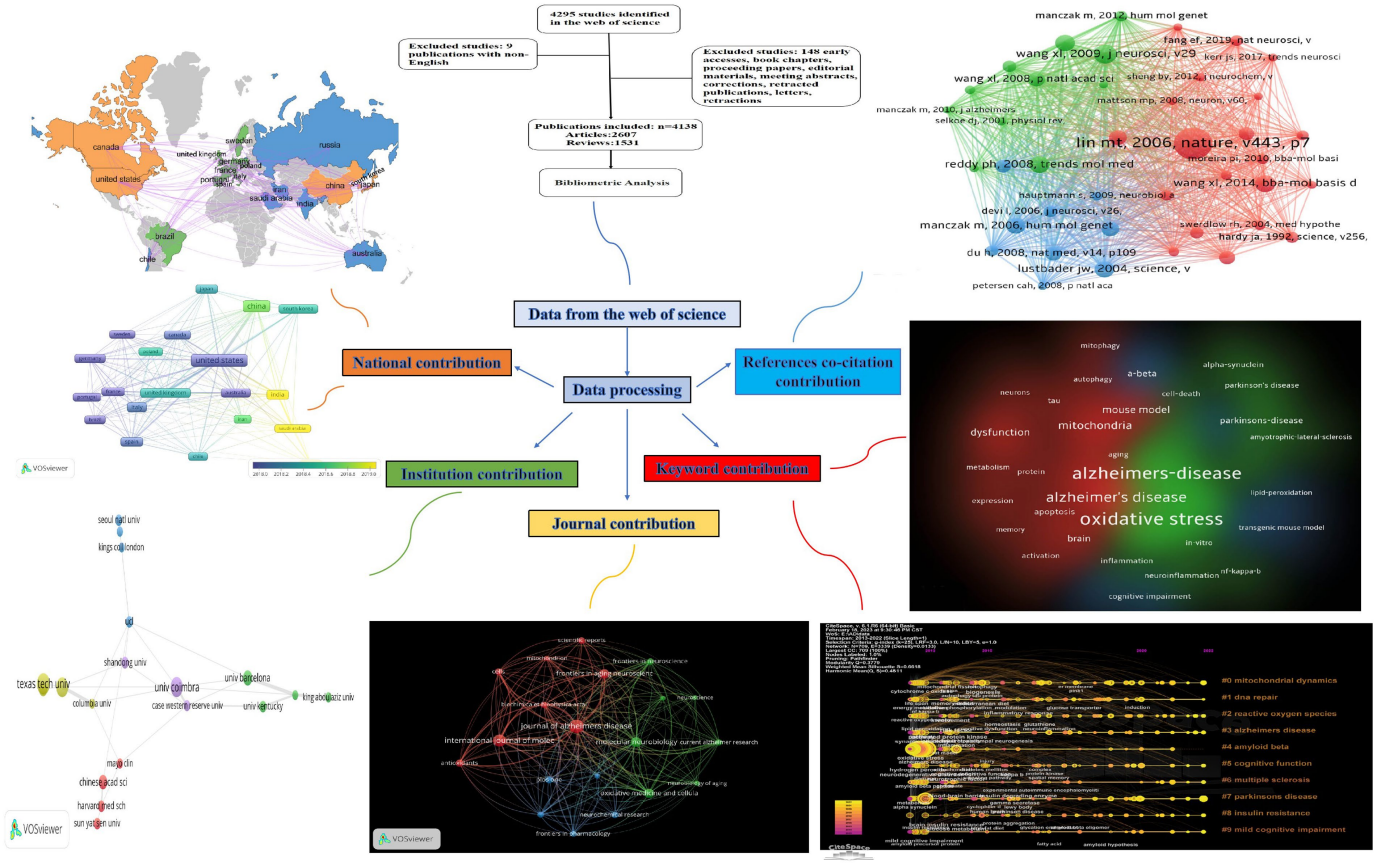
The flow diagram of the visual analysis with five aspects about mitochondrial dysfunction and AD. The contributions of different countries, different institutions, different journals, keywords, and references co-citation are listed.

We used the H-index as an indicator to assess scientific achievement (Hirsch, 2005), which was defined as at least H articles published by a researcher or a country, and each paper was cited at least H times by other publications. The H-index was calculated by RSstudio. IF was provided by the 2022 Journal Citation Report (JCR). VOSview or CiteSpace was used to analyze and visualize bibliometric data (van Eck and Waltman, 2017; Sui et al., 2019), such as countries, authors, journals, institutions, and keywords. The linkage strength map of the country and heatmap were jointly produced by VOSviewer and SCImago. Figure 2 illustrated our analysis process.

## Results

### Annual publication trends

In this study, we included 4,138 articles (9 non-English articles and 148 articles of type that did not match the requirements were exclued) published from 2013 to 2022 that fit our research theme.

**Figure 3:**
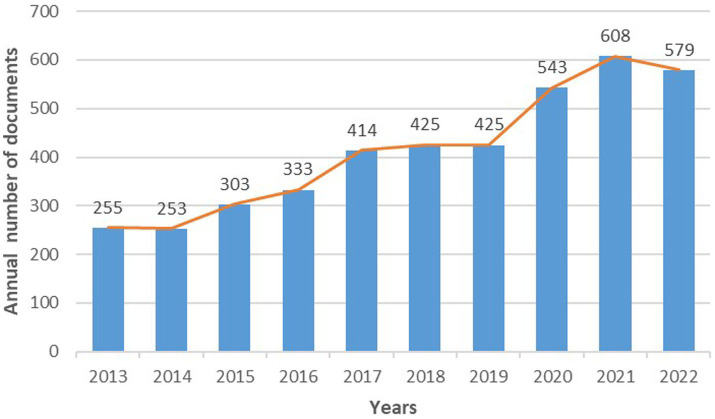
Annual publication trends in mitochondrial dysfunction and AD from 2013 to 2022.

Figure 3 suggests that the articles on Alzheimer’s disease and mitochondrial dysfunction generally tended to rise, but they began to decline in 2022. In 2021, 608 articles had been published, which was also the year with the most publications. However, it began to decline slightly in 2022 (579 publications). We present the characteristics of research data in five aspects: national contribution, institutional contribution, journal contribution, keyword contribution, and references co-citation contribution (Figure 3).

### Analysis of contributions from different countries

**Figure 4:**
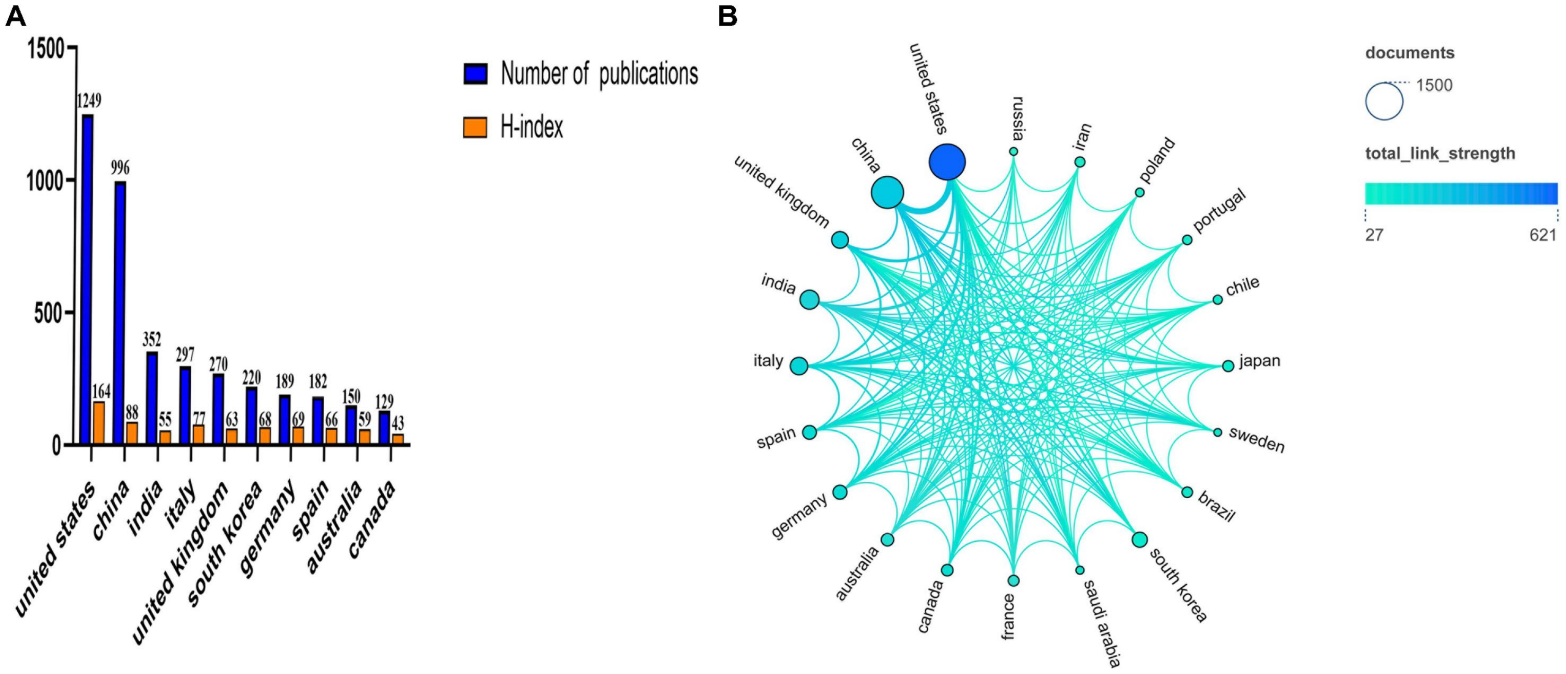
Contributions from different countries to research mitochondrial dysfunction and AD, and the collaborative networks between individual countries. **(A)** The number of publications and H-index in the top 10 countries. **(B)** Visualization of the number of publications and cooperation networks of the top 20 countries.

Our results show that these publications were shared with 90 different countries from 2013 to 2022. The top three countries with the number of articles were the United States, China, and India, which published 1,249, 996, and 352 articles, respectively. The number of articles issued by the United States and China was more than half of all publications (54.25%). Italy, South Korea, and the United Kingdom each published 200–300 articles. Germany, Spain, Australia, and other countries had less than 200 articles (Figure 4A). The country with the highest H-index was the United States (164), followed by China (88) and Italy (77). India had a higher volume of publications than Italy but occupied a lower H-index compared to Italy (Figure 4A). Figure 4B shows the intensity of cooperation between countries and the number of publications among the top 20 countries. The trend from green to blue means that the intensity of cooperation became stronger and stronger, using the circle size to indicate the number of publications. The thicker the connecting line, the closer the cooperation among countries in this field of research. The top three countries in terms of total link strength were the United States, China, and India, and the United States had the closest ties to China.

### Analysis of contributions from different institutions to publications

**Figure 5:**
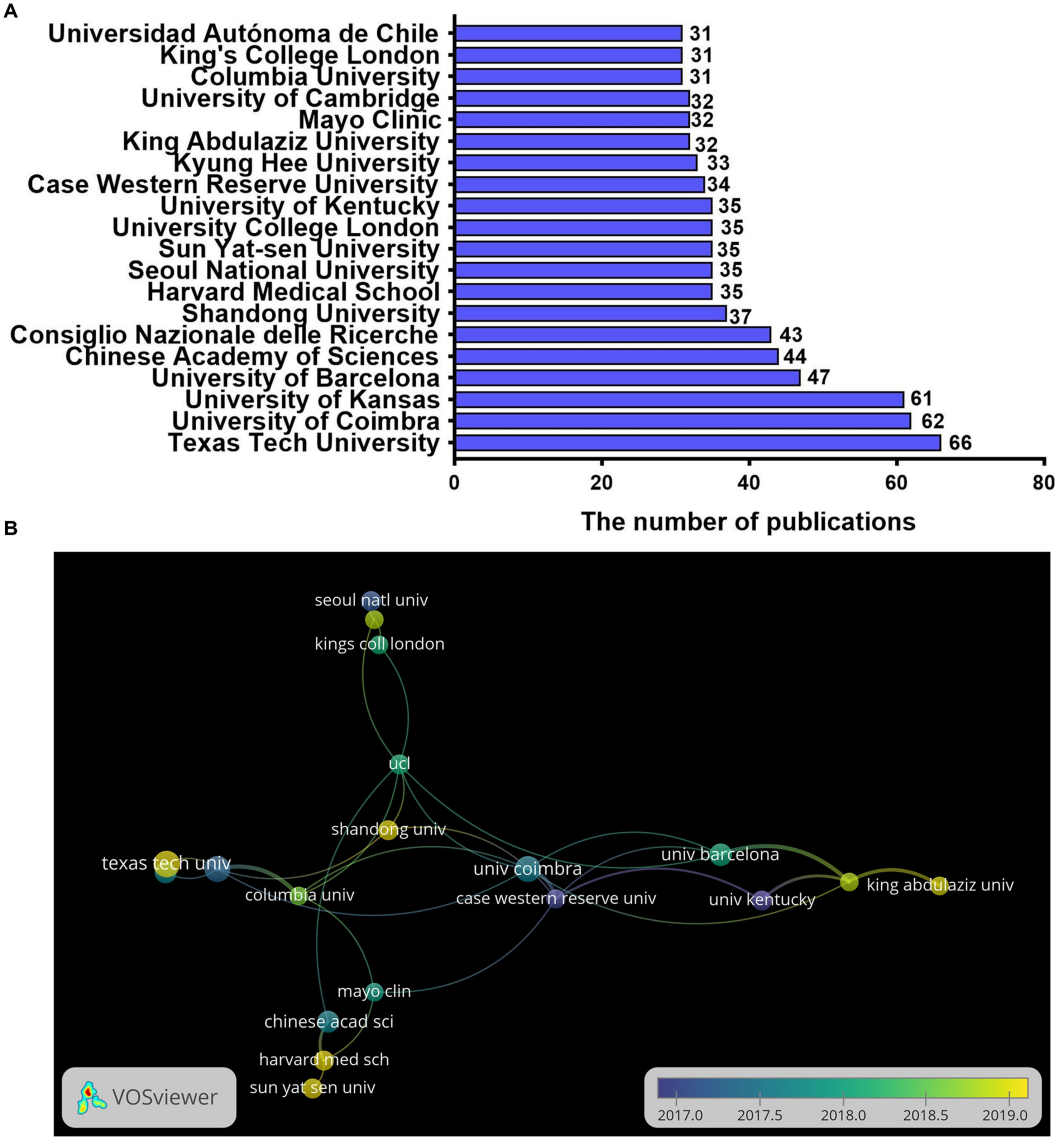
The distribution of institutions in research on mitochondrial dysfunction and AD. **(A)** Top 10 institutions by publishing volume. **(B)** Co-authorship overlay visualization map of the top 20 institutions produced in VOSviewer; the size of the circle represents the number of publications, the thickness of the connector represents the strength of the cooperation, and the color of the circle corresponds to the publication year.

The institution with the largest number of publications was Texas Tech University in the United States. Currently, the university has published 66 articles, accounting for 1.59% of all publications. The United States had occupied six positions in the top 20 institutions, with 263 articles published. In second and third place were the United Kingdom and China (Figure 5A). Figure 5B shows the connection between the volume of articles and the timeline, with yellow indicating the closest to the present time, and the size of the circle indicating the number of publications. The University of Kyung Hee had no cooperation with other institutions. The top three institutions with the number of publications were Texas Tech University, the University of Coimbra, and the University of Kansas. At present, the more active institutions are Shandong University, Texas Tech University, Harvard Medical School, King Abdulaziz University, and Sun Yat-sen University.

### Contribution of journals

**Figure 6:**
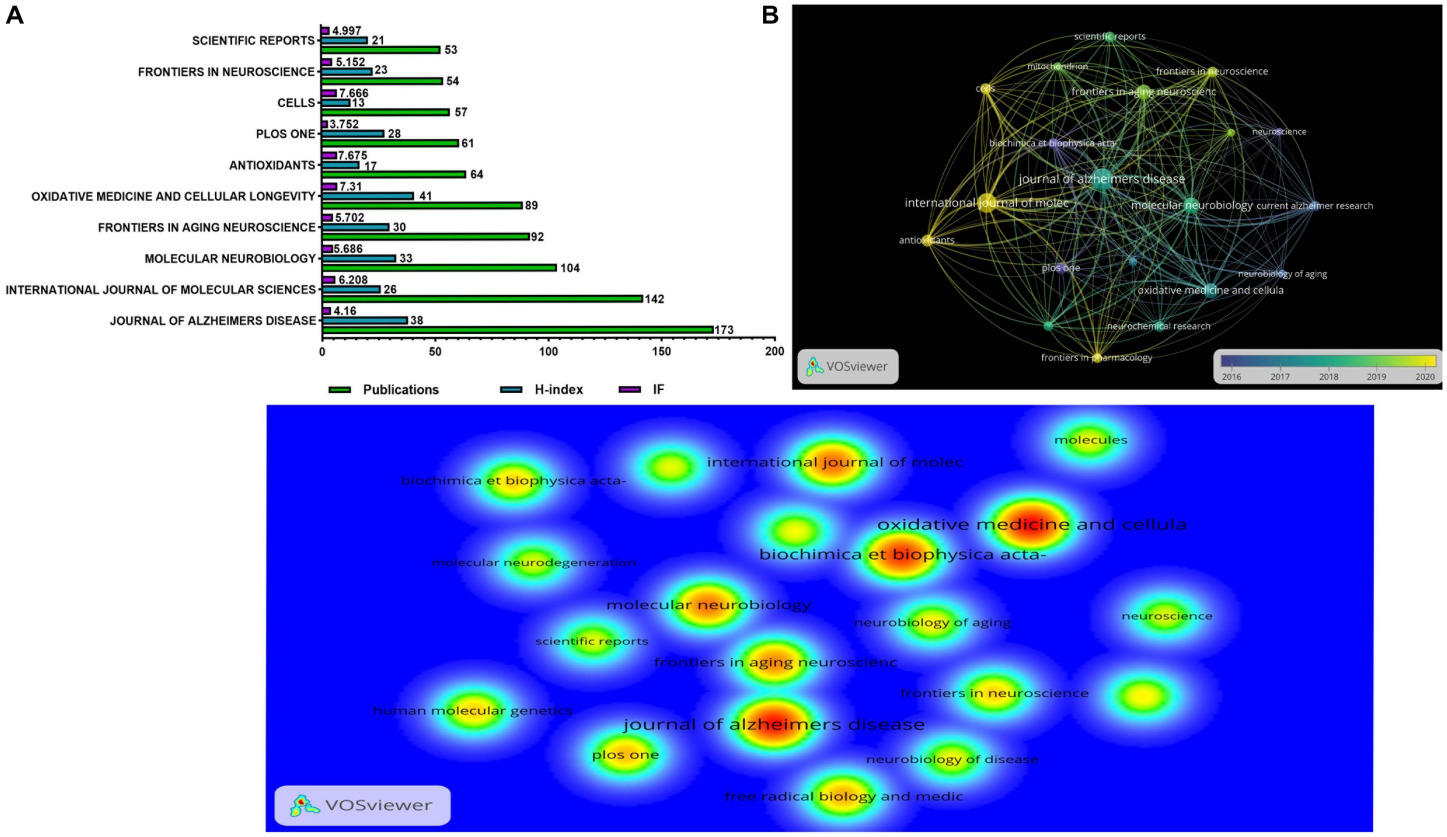
The distribution of journals engaged in research on mitochondrial dysfunction and AD. **(A)** The number of publications, H-index, and impact factor in the top 10 journals. **(B)** The citation network visualization map of the top 20 journals based on VOSviewer; the size of the circle represents the number of publications, the thickness of the connector represents the strength of the citation, and the color of the circle corresponds to the publication year. **(C)** The density visualization map of the cited top 20 institutions based on VOSviewer; the color blue to red indicates an increase in density.

In Figure 6A, the journal with the largest number of publications was the *Journal of Alzheimer’s Disease* (IF = 4.16; H-index = 38), which had 173 records. It was followed by the *International Journal of Molecular Sciences* (IF = 6.208, H-index = 26) and *Molecular Neurobiology* (IF = 5.686, H-index = 33) with 142 and 104 publications. The journals that ranked first in the H-index were *Oxidative Medicine and Cellular Longevity* (H-index = 41), followed by the *Journal of Alzheimer’s Disease* (H-index = 38) and *Molecular Neurobiology* (H-index = 33). Meanwhile, the journal with the highest impact factor was Cells (IF = 7.675). Figure 6B shows the connection between the number of publications and the timeline in different journals, with yellow indicating the closest to the present time and the size of the circle indicating the number of publications. Active was defined as more publications and closer to the current time. Currently, the most active journal is the *International Journal of Molecular Sciences,* and it also has more publications than other active journals. Figure 6C shows the citations of different journals, and the redder color indicated more citations. The journal with the highest number of citations was *Oxidative Medicine and Cellular Longevity* (5,790 citations), followed by the *Journal of Alzheimer’s Disease* (5,467 citations), and *Biochimica et Biophysica Acta-molecular Basis of Disease* (4,696 citations).

### Analysis of keywords

**Figure 7:**
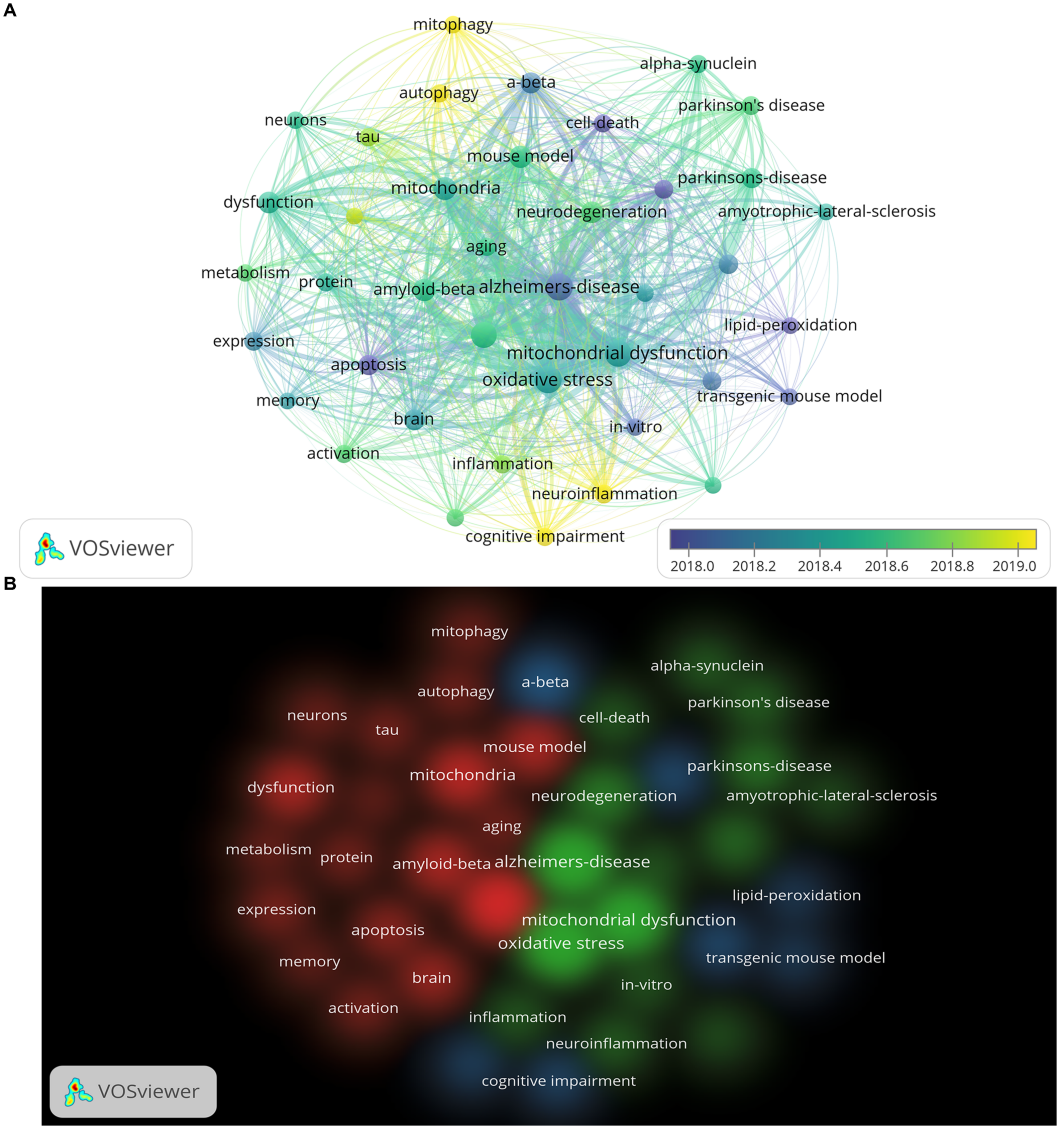
The visualization map of the top 40 keywords based on VOSviewer. **(A)** Keywords co-occurrence overlay visualization map; the size of the circle represents the frequency of occurrence per keyword, the thickness of the connector represents the strength of the connection between keywords, and the color of the circle corresponds to the publication year. **(B)** Clustering map of keywords based on VOSviewer; the same color represents the same cluster.

We extracted keywords with a minimum number of occurrences of 150 times from all publications through the VOSviewer. As a result, we got 40 keywords. The top 20 keywords are listed in Table 1. In this study, we list the top three keywords: oxidative stress (1,961 times), Alzheimer’s disease (1,848 times), and mitochondrial dysfunction (1,727 times). Figure 7A shows the top 40 keywords with frequency occurrences. These keywords were mainly from 2018 to 2019. The blue badge indicated the earliest occurrence, and the yellow was closer to the present time. Keywords marked in yellow might indicate new research directions, which deserved more attention. The keywords we found to be hot at the moment were: mitophagy, autophagy, neuroinflammation, and cognitive impairment. Figure 7B shows three different types of keyword clustering. Keywords of the same color had a stronger connection with each other. The red cluster contained keywords such as Alzheimer’s disease, mitochondria, dysfunction, and amyloid-beta. The green cluster contained keywords, such as oxidative stress, Alzheimer’s-disease, mitochondrial dysfunction, and neurodegeneration. The blue cluster contained keywords such as dementia, mild cognitive impairment, and cognitive impairment. These all indicated that mitochondrial dysfunction and oxidative stress were still the focus of research in this field.

**Table 1:** Top 20 co-occurrence times of keywords.

Ranking	Keywords	Frequency
1	Oxidative stress	1,961
2	Alzheimer’s-disease	1,848
3	Mitochondrial dysfunction	1,727
4	Alzheimer's disease	1,361
5	Mitochondria	813
6	Amyloid-beta	621
7	Mouse model	615
8	Dysfunction	610
9	Neurodegeneration	561
10	a-Beta	464
11	Brain	458
12	Parkinson’s-disease	428
13	Apoptosis	365
14	Neurodegenerative diseases	343
15	Amyloid precursor protein	288
16	Mild cognitive impairment	283
17	Parkinson's disease	272
18	Expression	269
19	Neuroinflammation	253
20	Activation	236

### Analysis of references

**Figure 8:**
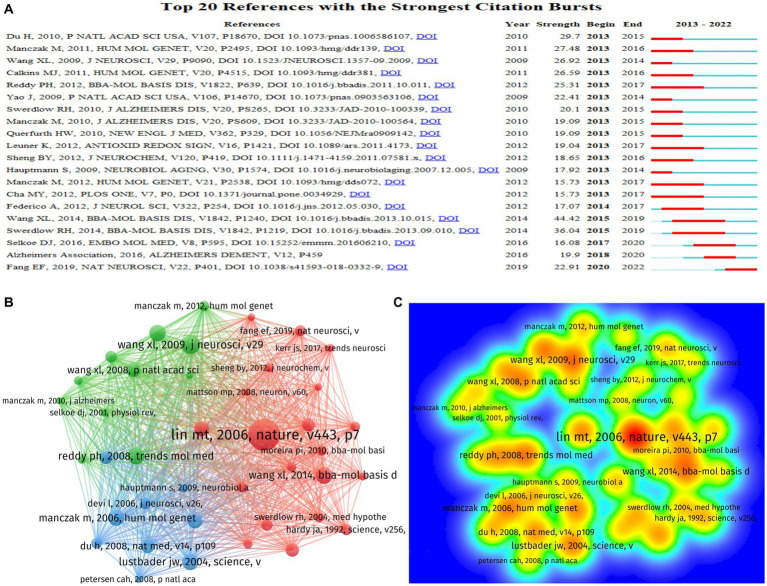
The distribution of references in research on mitochondrial dysfunction and AD. **(A)** The top 20 references with the strongest citation bursts based on CiteSpace; the red bars indicate the years with the highest frequent publications, and the green bars indicate the years with the lowest frequent publications. **(B)** The co-citation network visualization map of references based on VOSviewer. **(C)** The density visualization map of co-cited top 40 institutions based on VOSviewer; the color blue to red indicates an increase in density.

In the last 10 years, 218,637 references have been cited in this field’s publications, with an average of 53 citations per article. We presented the top 20 references with the strongest citation bursts through CiteSpace (Figure 8A). Figures 8B,C show the network and density of the top 40 co-cited references. The top 10 cited references are listed in Table 2, which contains four animal experiments, two experiments on human autopsy specimens, one cell experiment, and three reviews. The topics of these seven experiments were all about mitochondrial dysfunction, mainly related to oxidative damage, abnormal mitochondrial dynamics, and mitochondrial bioenergetic deficit. We found that mitophagy was a hotspot in current research, according to the analysis of citation bursts until 2022.

**Table 2:** Top 10 co-cited references.

Ranking	References	Co-cited	Author (year)
1	Mitochondrial dysfunction and oxidative stress in neurodegenerative diseases	529	Lin and Beal (2006)
2	Impaired balance of mitochondrial fission and fusion in Alzheimer’s disease	312	Wang X (2009)
3	Oxidative stress and mitochondrial dysfunction in Alzheimer’s disease	283	Wang X (2014)
4	Amyloid beta, mitochondrial dysfunction, and synaptic damage: implications for cognitive decline in aging and Alzheimer’s disease	281	Reddy PH (2008)
5	ABAD directly links Abeta to mitochondrial toxicity in Alzheimer’s disease	273	Lustbader JW (2004)
6	Impaired mitochondrial dynamics and abnormal interaction of amyloid beta with mitochondrial protein Drp1 in neurons from patients with Alzheimer’s disease: implications for neuronal damage	273	Manczak M (2011)
7	Mitochondria are a direct site of A beta accumulation in Alzheimer’s disease neurons: implications for free radical generation and oxidative damage in disease progression	267	Manczak M (2006)
8	Mitochondrial bioenergetic deficit precedes Alzheimer’s pathology in female mouse models of Alzheimer’s disease	249	Yao J (2009)
9	Mitochondrial abnormalities in Alzheimer’s disease	248	Hirai K (2001)
10	Amyloid-beta overproduction causes abnormal mitochondrial dynamics via differential modulation of mitochondrial fission/fusion proteins	236	Wang X (2008)

## Discussion

This study has used bibliometric methods to evaluate mitochondrial dysfunction and AD, and it will provide researchers with research hotspots and trends in this field. We got 4,138 articles in this field from WOS. The number of publications can reflect the productivity and developments in a particular field of study (Durieux and Gevenois, 2010). Annual publication trends show that there was more and more research on mitochondrial dysfunction and Alzheimer’s disease from 2013 to 2021. However, the number of publications in 2022 was lower than in 2021, but still more than in 2019 and previous years. In the future, research on Alzheimer’s disease and mitochondrial dysfunction may be declining. The results showed the number of publications maintained at a high level in the past 3 years, which indicates that mitochondrial dysfunction remains a hot area in AD research.

The number of publications is an important indicator for evaluating the scientific research level of a country, institution, or author. The H-index is a useful indicator for evaluating the quality of an article, and it’s high score indicates the higher level of quality and recognition (Joshi, 2014). Our findings show that the United States and China are the two countries with the most publications in this field (Figure 4A). At the same time, they also have the highest H-index. From 2013 to 2022, international cooperation has become closer, and the United States remains the country with the highest level of cooperation. Especially, it cooperates with China, the United Kingdom, and India (Figure 4B). This also shows that mutual cooperation promotes development in the field. Among the top 20 research institutions, the United States also has the most research institutions (Figure 5A). The highest output is from Texas Tech University in the United States. Currently, the university is also one of the most active universities in this field of research. Other active institutions include Shandong University, Harvard Medical School, King Abdulaziz University, and Sun Yat-sen University (Figure 5B). These institutions also deserve our attention.

Through the analysis of journals, we found that most journals had an impact factor greater than 5 (Figure 6A). The *Journal of Alzheimer’s Disease* has the largest number of articles, but its H-index is lower than the Journal of *Oxidative Medicine and Cellular Longevity*. We should focus on journals with a high H-index and high output.

Currently, the most active journal is *International Journal of Molecular Sciences*. At the same time, the journals that have recently published more articles include Frontiers in Aging Neuroscience, *Antioxidants and Cells* (Figure 6B). The most cited journals are the *Journal of Alzheimer’s Disease and Oxidative Medicine and Cellular Longevity* (Figure 6C). The research content in these hot journals mainly includes genetic factors for mitochondrial dysfunction (Awasthi et al., 2021), mitochondrial damage caused by Aβ (Olajide et al., 2022), abnormalities in calcium signaling (Rigotto et al., 2021), and abnormal mitophagy (Reddy and Oliver, 2019). These results of bibliometric analyses can support researchers’ access to literature and submissions in the field (Oelrich et al., 2007).

Analyzed by keywords, we found that oxidative stress, mitochondrial dysfunction, Amyloid-Beta, autophagy, mitophagy, and neuroinflammation are focus areas of research in the past 10 years (Table 1). The hot keywords in this field are autophagy, mitochondrial autophagy, neuroinflammation, and cognitive impairment after 2019 (Figure 7A). These show that current hotspots are still focused on basic research in the field. Autophagy, oxidative stress, apoptosis, amyloid-β protein, endoplasmic reticulum, etc. are considered to be current research hotspots in this field by Song et al. (2023). It is similar to our research results, but our results suggested that more research focused on autophagy, mitochondrial autophagy, and neuroinflammation after 2019. At present, these hot keywords should be more worthy of researchers’ attention. Mitochondria is the source of energy produced for cells, and its functions include providing ATP, calcium buffering, hormone synthesis, reactive oxygen species (ROS) production, and participation in apoptosis (Battogtokh et al., 2018). As a type of cell that is extremely sensitive to energy requirements, neurons receive obvious effects from mitochondria. Disorders of the respiratory chain decreased ATP production, and increased ROS production have been demonstrated in AD (Area-Gomez et al., 2018). Low glucose metabolism of the brain was found in AD patients, which were examined by positron emission tomography (Adlimoghaddam et al., 2019). This has also been confirmed in animal experiments (Adlimoghaddam et al., 2019). Electron microscopy observed that mitochondria in neurons were attacked by Aβ (Ashleigh et al., 2023). Aβ has been shown to induce apoptosis in neurons (Maj et al., 2017). It has been found to cause cytotoxicity due to increased concentration of calcium in the mitochondria of the microglia (Xie et al., 2017). This may be due to the calcium permeation pores created by Aβ to the plasma membrane (Supnet and Bezprozvanny, 2010). Aβ and its isomers can also cause increased ROS, nitric oxide, and glutathione, leading to its dysfunction (Petrovskaya et al., 2022). In addition, studies have found that Aβ can affect mitochondrial function by regulating mitochondria-ER contact sites. In summary, mitochondrial dysfunction is still an important research direction in the pathogenesis of Alzheimer’s disease.

Autophagy and mitochondrial autophagy are also hotspots in the research of AD. Autophagy is a protective mechanism in the body that removes damaged organelles and abnormal proteins. Studies have shown the presence of autophagy abnormalities in AD, accompanied by neuroinflammation, clearance defects, Aβ and Tau deposition, and synaptic dysfunction (Ou-Yang et al., 2023). The study has found that mitochondrial autophagy is reduced by 60% in the hippocampus of AD model mice (Fang et al., 2019). Mitochondrial autophagy is considered a selective type of autophagy, and abnormal mitochondria will be removed this way (Kalani et al., 2023). These abnormal mitochondria are blocked in autophagosomes and further degraded by lysosomes (Cai and Tammineni, 2016). Mitochondrial autophagy can be mediated by ubiquitin, lipid, Parkin, and receptor (Kalani et al., 2023). Some researchers have found that the use of autophagy inducers to act on BV-2 cells can reduce mitochondrial apoptosis, ROS production, and neuroinflammation (Lei et al., 2018). Similar results are found in a mice AD model (Hou et al., 2018). When the antioxidant system is destroyed, ROS expression increases, resulting in the mitochondria suffering from oxidative damage and destroying the enzyme function in the respiratory chain (Blagov et al., 2022). Eventually, this leads to mitochondrial dysfunction. Glial cell activation has been observed in patients with AD, and it has been characterized as neuroinflammation. Activated glial cells initiate a cascade of inflammatory responses surrounding senile plaques (Heneka et al., 2015). A new type of activated microglia has been discovered and named disease-associated microglia in mice. They are found to be closely related to Aβ deposition (Sobue et al., 2023). However, the mechanism underlying these findings is intricate and warrants further exploration. The role of glial cells in AD pathogenesis, particularly in the context of neuroinflammation and autophagy, is expected to emerge as a prominent research focus in the future.

The treatment of AD remains a formidable challenge in the field. Some studies have found that mitochondrial-targeted antioxidants can be a new method for the treatment of AD, and the use of mitochondrial-targeted micelles nanocarriers can effectively reduce ROS in mitochondria and reduce oxidative stress (Oliver and Reddy, 2019; Yang et al., 2020). We found that the article of Lin MT is the most cited by analyzing references (Lin and Beal, 2006). This is a review published in Nature that supports many subsequent studies (Figure 8C). The top 10 cited references are all more than 8 years old (Table 2). This shows that earlier studies still make a clear contribution to current research, and researchers should use them as references.

Currently, researchers are actively engaged in exploring the mechanisms and treatments associated with mitochondrial dysfunction in AD. This study, utilizing bibliometric analysis, provides a comprehensive summary of the prevailing research trends and hotspots concerning mitochondrial dysfunction in AD. These findings serve as a valuable guide for researchers to navigate the current research landscape. It is our anticipation that these endeavors will lead to significant advancements in this field.

## Limitations

We used bibliometric methods to analyze the last 10 years of studies of mitochondrial dysfunction and AD. This is the first bibliometric evaluation in this field. However, there are still some shortcomings in our study. For example, our data came from WOS and are limited to English, and it may not be exhaustive. Overall, the research trends in this field will not change too significantly.

## Conclusion

In our study, we used VOSviewer1.6.18 and CiteSpace 6.1. R6 to evaluate the research status and trends in mitochondrial dysfunction and AD over the past 10 years. The United States and China have made major contributions in this area. International cooperation is very important. It is expected to expand in the future. In the rankings of publication volume, Texas Tech University and the *Journal of Alzheimer’s Disease* topped the list. In the citation ranking, the most cited are *Oxidative Medicine and Cellular Longevity* and Lin MT’s research. The molecular mechanism of mitochondrial dysfunction in AD is still a hotspot in the future. These results will help researchers pay attention to the direction and hotspots of this research field.

## Data availability statement

The original contributions presented in the study are included in the article/supplementary material; further inquiries can be directed to the corresponding author.

## Author contributions

YL and H-xZ designed the study. The data were analyzed by WG and LH. Y-hC, X-pY, and S-xW checked the data. The manuscript was written by WG and was reviewed by LH and YL. All authors contributed to the article and approved the submitted version.

## Conflict of interest

The authors declare that the research was conducted in the absence of any commercial or financial relationships that could be construed as a potential conflict of interest.

## Publisher’s note

All claims expressed in this article are solely those of the authors and do not necessarily represent those of their affiliated organizations, or those of the publisher, the editors and the reviewers. Any product that may be evaluated in this article, or claim that may be made by its manufacturer, is not guaranteed or endorsed by the publisher.
